# Knockdown of PTK7 Reduces the Oncogenic Potential of Breast Cancer Cells by Impeding Receptor Tyrosine Kinase Signaling

**DOI:** 10.3390/ijms241512173

**Published:** 2023-07-29

**Authors:** Won-Sik Shin, Si Won Oh, Han Na Park, Jae Hoon Kim, Seung-Taek Lee

**Affiliations:** Department of Biochemistry, College of Life Science and Biotechnology, Yonsei University, Seoul 03722, Republic of Korea; amgod0306@hanmail.net (W.-S.S.); siwon1993@gmail.com (S.W.O.); qkrgkssk1024@naver.com (H.N.P.); ebahspa@yonsei.ac.kr (J.H.K.)

**Keywords:** protein tyrosine kinase 7 (PTK7), receptor tyrosine kinase (RTK), breast cancer (BC), triple-negative breast cancer (TNBC), targeted therapy

## Abstract

Protein tyrosine kinase 7 (PTK7), a catalytically defective receptor tyrosine kinase (RTK), is often upregulated in various cancers. This study aimed to validate PTK7 as a target for breast cancer (BC) and investigate its oncogenic signaling mechanism. BC tissue analysis showed significantly elevated PTK7 mRNA levels, especially in refractory triple-negative breast cancer (TNBC) tissues, compared with normal controls. Similarly, BC cell lines exhibited increased PTK7 expression. Knockdown of PTK7 inhibited the proliferation of T-47D and MCF-7 hormone-receptor-positive BC cell-lines and of HCC1187, MDA-MB-231, MDA-MB-436, and MDA-MB-453 TNBC cells. PTK7 knockdown also inhibited the adhesion, migration, and invasion of MDA-MB-231, MDA-MB-436, and MDA-MB-453 cells, and reduced the phosphorylation levels of crucial oncogenic regulators including extracellular signal-regulated kinase (ERK), Akt, and focal adhesion kinase (FAK). Furthermore, PTK7 interacts with fibroblast growth factor receptor 1 (FGFR1) and epidermal growth factor receptor (EGFR) expressed in MDA-MB-231 cells. Knockdown of PTK7 decreased the growth-factor-induced phosphorylation of FGFR1 and EGFR in MDA-MB-231 cells, indicating its association with RTK activation. In conclusion, PTK7 plays a significant role in oncogenic signal transduction by enhancing FGFR1 and EGFR activation, influencing BC tumorigenesis and metastasis. Hence, PTK7 represents a potential candidate for targeted BC therapy, including TNBC.

## 1. Introduction

Breast cancer (BC) is the most common cancer among women, and is a major cause of morbidity and mortality in women worldwide [1]. BC is diagnosed in 12.4% of women in the United States at some point in their life; in 2017, more than 250,000 new cases of BC were diagnosed in women in the United States [2].

BC is a complex disease that exhibits a high degree of inter- and intratumoral heterogeneity [3]. For convenience, BC can be divided into three main subtypes: (1) estrogen receptor (ER)-positive and progesterone receptor (PR)-positive and epidermal growth factor receptor 2 (HER2)-negative (70% of patients); (2) HER2-positive (15–20%); and (3) triple-negative, i.e., negative for all three markers (15%) [2]. Triple-negative breast cancer (TNBC) predominantly occurs in young premenopausal women aged under 40 years [4]. Patients with TNBC have a 40% mortality rate within the first 5 years after diagnosis [5], and a shorter average survival than those with other subtypes of BC. TNBC is highly invasive, and approximately 46% of patients with TNBC have distant metastasis. The average survival time after metastasis is only 13.3 months and the recurrence rate after surgery is 25% [6].

Protein tyrosine kinase 7 (PTK7) is a receptor tyrosine kinase (RTK) that contains an extracellular domain with seven immunoglobulin-like loops, a transmembrane domain, and a defective tyrosine kinase domain that exhibits no catalytic activity [7,8,9]. Mice expressing a truncated form of PTK7 polypeptide showed perinatal death with a defect in neural-tube closure and stereociliary-bundle orientation, suggesting that PTK7 is a regulator of planar-cell polarity (PCP) [10]. The interaction between PTK7 and Dsh proteins at the plasma membrane activates non-canonical Wnt signaling, which then directs PCP [11]. PTK7 interacts with β-catenin [12] or maintains LDL receptor-related protein 6 stability [13], thereby enhancing the Wnt/β-catenin pathway. These findings suggest that PTK7 plays roles in the canonical Wnt pathway, the non-canonical Wnt pathway, and the PCP signaling pathway, at least during embryonic development.

Increased PTK7 expression in diverse types of cancer has been reported, as has an association between the expression of PTK7 and tumorigenesis. The expression of PTK7 in leukemia cells enhances cell migration and survival [14]. PTK7 knockdown reduces the proliferation and anti-apoptotic activity of colon cancer cells [15] and inhibits the proliferation, migration, and invasion of liposarcoma cells [16]. PTK7 is associated with proliferation, migration, and invasiveness, and activates FGFR1 independently of FGF in esophageal squamous cell carcinoma (ESCC) cells [17,18,19]. Impairment of the PTK7 function prevents the vascular endothelial growth factor (VEGF)-induced migration, invasion, and tube-formation of human umbilical vein endothelial cells (HUVECs), and angiogenesis in vivo by reducing kinase insert domain receptor (KDR) activation [20,21].

Knockdown of PTK7 mediated by siRNA, treatment with an anti-PTK7 polyclonal antibody, or overexpression of a kinase domain-deleted PTK7 mutant have been shown to decrease the motility and invasiveness of ER-positive Hs578T BC cells [22]. Patients with PTK7-negative tumors show better disease-free survival than those with PTK7-positive tumors, particularly with anthracycline treatment, suggesting that PTK7 is involved in resistance to anthracycline-based chemotherapy [23]. PTK7 expression is associated with tumor size and lymph-node metastasis in BC [22]. PTK7 is enriched in tumor-initiating cells from patient-derived xenografts of TNBC, ovarian cancer, and non-small-cell lung cancer [24]. Overexpression or knockdown of PTK7 increases or decreases the tumorigenicity of ESCC KYSE-30 cells, respectively [25]. The administration of PTK7 neutralizing monoclonal antibodies resulted in a reduction in cellular and in vivo tumorigenicity in ESCC [26]. Additionally, these monoclonal antibodies demonstrate inhibitory effects on angiogenesis in vitro, ex vivo, and in vivo by blocking the interaction between PTK7 and KDR [27]. Furthermore, PTK7-targeted antibody–drug conjugates exhibited a decrease of tumor-initiating cells and induced tumor regression [24]. 

The potential role of PTK7 in tumorigenesis has been documented in different cancers, including BC. To date, however, the molecular mechanism of PTK7 in BC progression is poorly understood. To assess the potential value of PTK7 in the progression of BC, including TNBC, we analyzed the expression of *PTK7* mRNA expression using The Cancer Genome Atlas (TCGA) database for tumor and adjacent non-tumor tissue samples of BC patients. In addition, we analyzed the effect of PTK7 knockdown on the oncogenic properties of BC cell lines and on PTK7-mediated signaling pathways in TNBC cell lines.

## 2. Results

### 2.1. PTK7 Expression Is Upregulated in BC Tissues and Cell Lines

**Figure 1:**
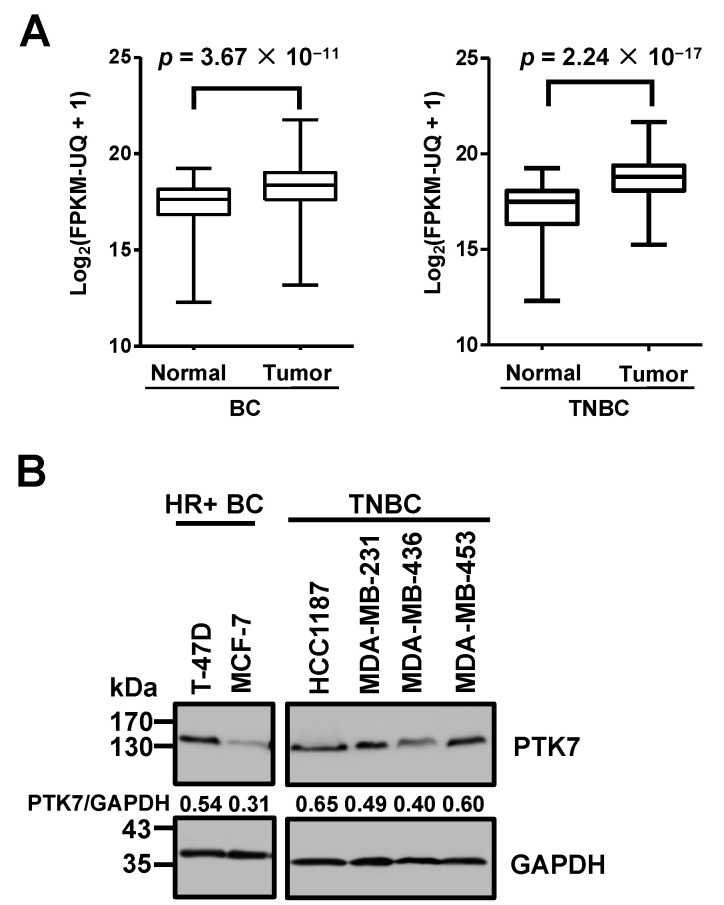
Expression of PTK7 in breast cancer (BC) tissues and cell lines. (**A**) Box–whisker plots showing *PTK7* mRNA levels in normal and tumor tissues of BC (**left**) and in triple-negative breast cancer (TNBC) (**right**) included in the database from The Cancer Genome Atlas (TCGA). (**B**) Western blot showing PTK7 expression levels in hormone-receptor-positive (HR+) BC and TNBC cell lines. GAPDH levels are displayed for normalization. The numbers below the PTK7 panels indicate the densitometric values of PTK7 bands relative to the corresponding GAPDH bands.

Our previous expression profile analysis in various cancer types using RNA-seq data from the TCGA database showed that the median level of *PTK7* mRNA [Log_2_ (FPKM-UQ + 1) = 18.37] in BC tissues was higher than the overall mean [Log_2_ (FPKM-UQ + 1) = 17.92], where FPKM-UQ, as explained in the Methods, is the upper quartile of fragments per kilobase of transcript per million mapped reads [28]. Analysis of the 1097 tumor tissues and 113 normal tissues of BC in the TCGA database showed that the median level of *PTK7* mRNA, Log_2_ (FPKM-UQ + 1), in tumor tissues (18.37) was significantly higher than that in normal tissues (17.64; *p* = 3.67 × 10^−11^; Figure 1A, left panel). In TNBC, the median level of *PTK7* mRNA, Log_2_ (FPKM-UQ + 1), in 430 tumor tissues (18.80) was significantly higher than the median level in 90 normal tissues (17.50; *p* = 2.24 × 10^−17^; Figure 1A, right panel). Western blot analysis revealed that PTK7 polypeptide was highly expressed in ER- and PR-positive (hormone-receptor-positive; HR+) T-47D BC cells and HCC1187, MDA-MB-231, MDA-MB-436, and MDA-MB-453 TNBC cells (Figure 1B).

### 2.2. Knockdown of PTK7 Reduces Proliferation of BC Cells

**Figure 2:**
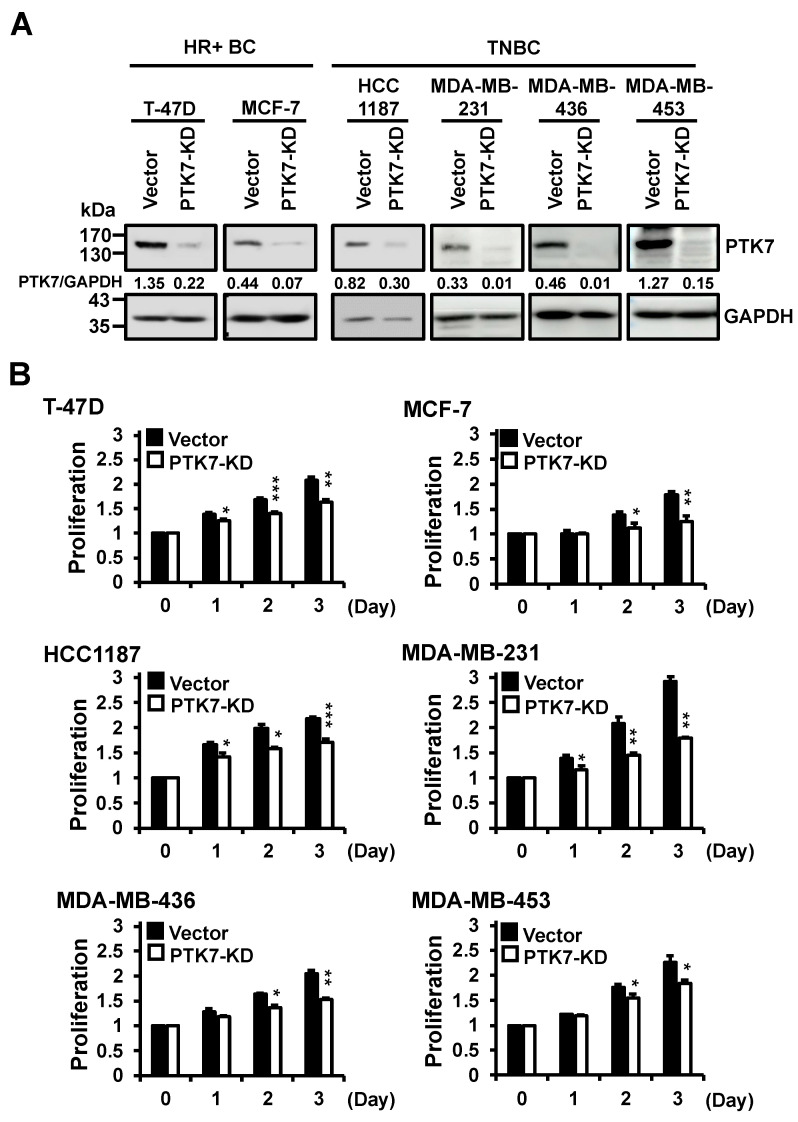
Effect of PTK7 knockdown on the proliferation of BC cells. (**A**) The level of PTK7 polypeptide in BC cells infected with lentiviruses for control (vector) and *PTK7* knockdown shRNA (PTK7-KD) was analyzed by Western blotting. GAPDH levels are displayed for normalization. The numbers below the PTK7 panels indicate the densitometric values of PTK7 bands relative to the corresponding GAPDH bands. (**B**) Proliferation of the control (vector) and PTK7-KD BC cells was analyzed for 3 days. Each bar represents the mean ± standard deviation (SD) from three independent experiments. * *p* < 0.05, ** *p* < 0.01, and *** *p* < 0.001 vs. control vector.

To examine the effect of PTK7 downregulation on the oncogenic properties of BC cells, PTK7 was silenced in T-47D and MCF-7 HR-positive BC cells and HCC1187, MDA-MB-231, MDA-MB-436, and MDA-MB-453 TNBC cells by infection with a lentivirus encoding *PTK7* shRNA (Figure 2A). Using PTK7-knockdown BC cells, we analyzed the effect of PTK7 on cell proliferation using a 3-(4,5-dimethylthiazol-2-yl)-2,5-diphenyltetrazolium bromide (MTT) assay. Knockdown of PTK7 reduced cell proliferation to 79% and 70% in T-47D and MCF-7 HR-positive BC cells, respectively, compared with the control vector, after 3 days of culture (Figure 2B). Knockdown of PTK7 also decreased cell proliferation to 79%, 62%, 75%, and 81%, respectively, in HCC1187, MDA-MB-231, MDA-MB-436, and MDA-MB-453 TNBC cells (Figure 2B). Additionally, to investigate whether PTK7 levels influence cell proliferation at the microscopic level, we conducted immunostaining of MDA-MB-231 cells, representing one of the breast cancer cell lines, using an antibody against Ki-67, a well-known marker for cell proliferation. The results demonstrated that knockdown of PTK7 led to a significant reduction in nuclear Ki-67 levels to 46.8 ± 0.5% (Supplementary Figure S1). These findings demonstrate that knockdown of PTK7 significantly reduced cell proliferation not only in HR-positive BC cells, but also in TNBC cells. 

### 2.3. Knockdown of PTK7 Decreases Cell Adhesion, Migration, and Invasion of TNBC Cells

**Figure 3:**
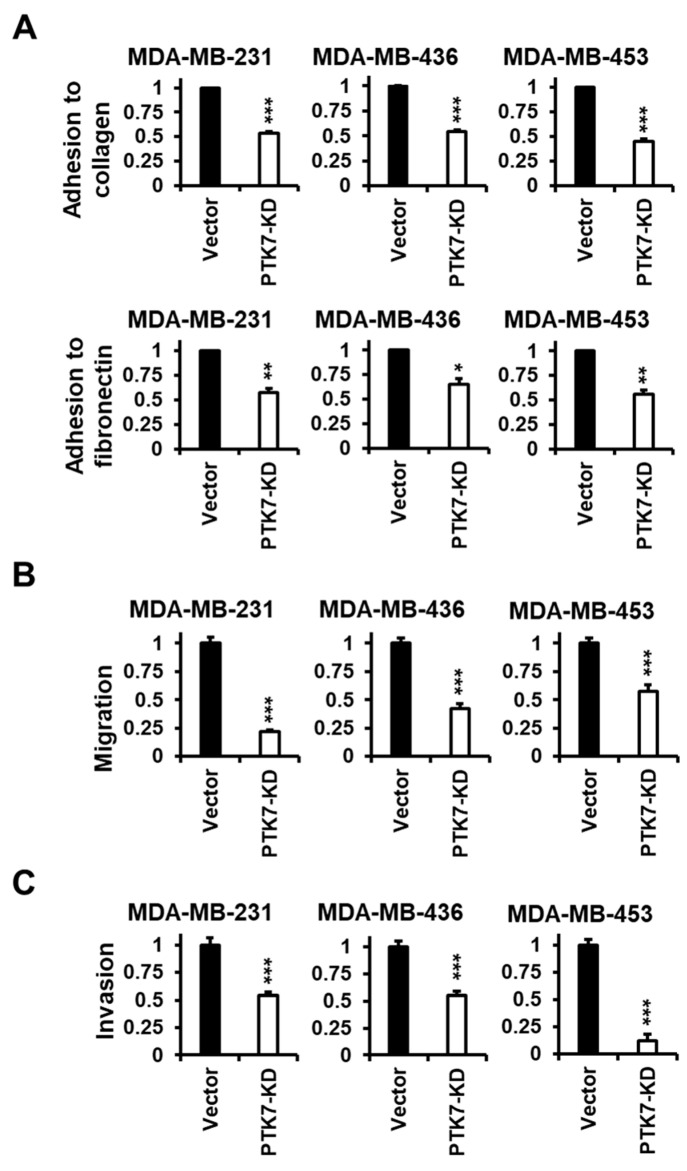
Effect of PTK7 knockdown on the adhesion, migration, and invasion of TNBC cells. (**A**) Adhesion of the control (vector) and PTK7-KD MDA-MB-231, MDA-MB-436, and MDA-MB-453 cells was analyzed at 2 h after plating on collagen-coated or fibronectin-coated dishes. (**B**) Chemotactic migration of the control (vector) and PTK7-KD cells was analyzed for 24 h. (**C**) Invasion of the control (vector) and PTK7-KD cells was analyzed for 48 h. Each bar represents the mean ± standard deviation (SD) from three independent experiments. * *p* < 0.05, ** *p* < 0.01, and *** *p* < 0.001 vs. control vector.

Although the anti-proliferative effects of PTK7 knockdown were very similar in HR-positive BC cells and in TNBC cells, our investigation focused on cells of TNBC, which generally has poorer prognoses and is more aggressive. Therefore, we analyzed the effect of PTK7 knockdown on cell adhesion, migration, and invasion in TNBC cells. Knockdown of PTK7 significantly reduced adhesion to a collagen-coated surface to 53%, 54%, and 45% of control, and adhesion to a fibronectin-coated surface to 57%, 65%, and 56% in MDA-MB-231, MDA-MB-436, and MDA-MB-453 cells, respectively (Figure 3A). Compared with control vectors, knockdown of PTK7 also reduced cell migration, measured using a gelatin-coated Boyden chamber, to 22%, 42%, and 58% in MDA-MB-231, MDA-MB-436, and MDA-MB-453 cells, respectively (Figure 3B). The invasion of PTK7-knockdown cells decreased to 54%, 55%, and 12% in MDA-MB-231, MDA-MB-436, and MDA-MB-453 cells, respectively, compared with control cells (Figure 3C). From these results, we confirmed that knockdown of PTK7 significantly reduced the oncogenic phenotypes of TNBC cells.

### 2.4. PTK7 Knockdown Inhibits the Activation of ERK, Akt, and FAK in TNBC Cells

**Figure 4:**
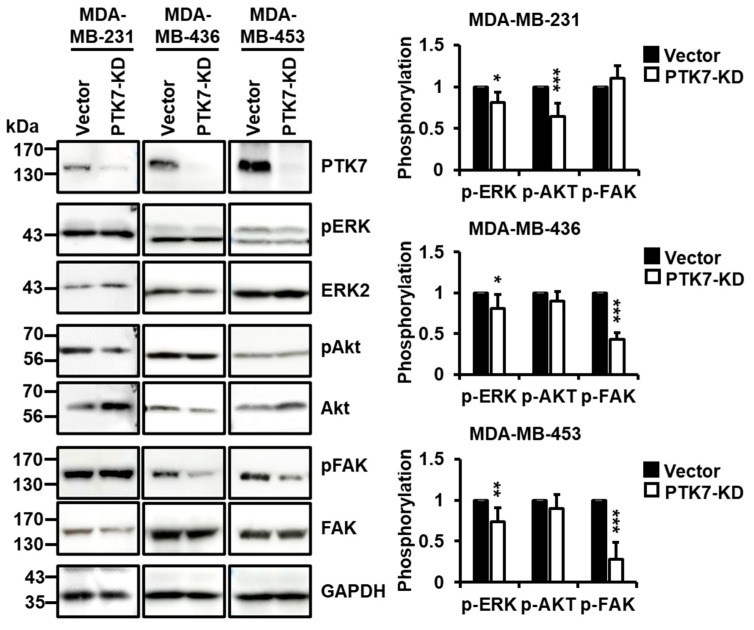
Effect of PTK7 knockdown on the activation of ERK, Akt, and FAK signaling in TNBC cells. The phosphorylation of ERK, Akt, and FAK was analyzed by Western blotting at 16 h after replacement with fresh complete medium in the control (vector) and PTK7-KD MDA-MB-231, MDA-MB-436, and MDA-MB-453 cells. GAPDH levels are displayed for normalization. Each bar represents the mean ± standard deviation (SD) from three independent experiments. * *p* < 0.05, ** *p* < 0.01, and *** *p* < 0.001 vs. control vector.

Activation of ERK and Akt is crucial for oncogenic phenotypes, such as cell proliferation and survival in various cancers, including BC [29]. In addition, FAK activation is important for cell adhesion, migration, and invasion [30,31]. Accordingly, we analyzed the role of PTK7 in the activation of ERK, Akt, and FAK. In MDA-MB-231 cells, knockdown of PTK7 significantly decreased the phosphorylation levels of ERK and Akt. Additionally, in MDA-MB-436 and MDA-MB-453 cells, the knockdown of PTK7 significantly decreased the phosphorylation levels of ERK and FAK (Figure 4). These results suggest that PTK7 activates signaling pathways mediating oncogenic phenotypes.

### 2.5. PTK7 Interacts with FGFR1, EGFR, and ROR2 in HEK293 Cells

**Figure 5:**
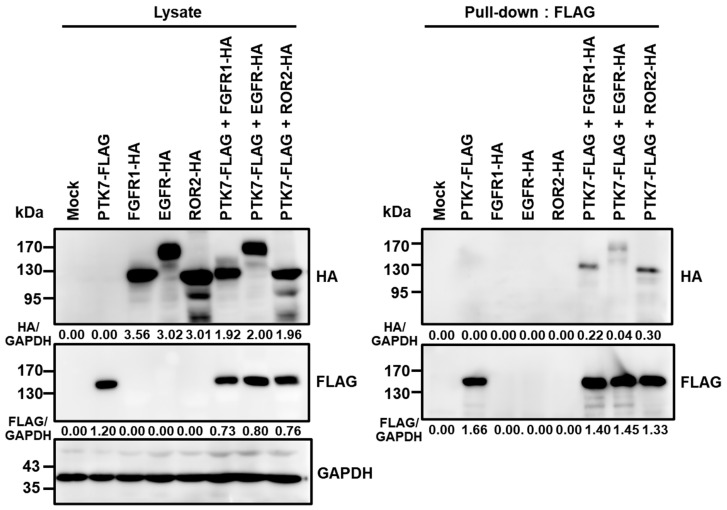
Binding of PTK7 with FGFR1, EGFR, and ROR2 in HEK293 cells. The binding of PTK7 to FGFR1, EGFR, or ROR2 in HEK293 cells co-expressing PTK7-FLAG and FGFR1-HA, EGFR-HA, or ROR2-HA was analyzed. Cell lysates were pulled down with FLAG. The numbers below the HA or FLAG panels indicate the densitometric values of HA or FLAG bands relative to the corresponding GAPDH bands.

Previous studies have reported the interaction between PTK7 and ROR2, FGFR1, or EGFR in cancer cells [19,32]. To further explore the binding of these RTKs to PTK7, pull-down assays were performed using HEK293 cells co-expressing PTK7 along with either FGFR1, EGFR, or ROR2. The results showed that PTK7 could bind to all three analyzed RTKs, FGFR1, EGFR, and ROR2 (Figure 5). However, judging by the amount of RTK-HA co-precipitated with PTK7-FLAG, the order of interaction with PTK7 was ROR2, FGFR1, and EGFR (Figure 5).

### 2.6. PTK7 Knockdown Inhibits the Activation of FGFR1 and EGFR in MDA-MB-231 Cells

**Figure 6:**
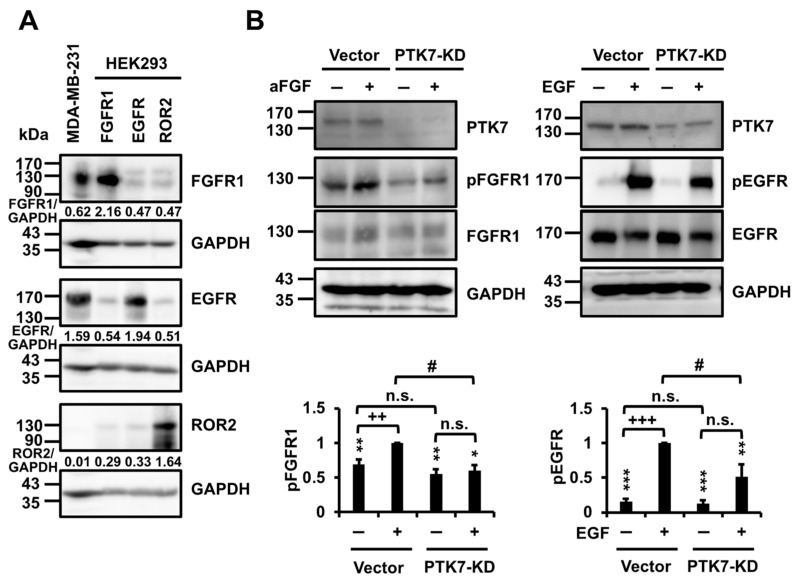
Effect of PTK7 knockdown on the phosphorylation of FGFR1 and EGFR in MDA-MB-231 cells. (**A**) The expression levels of FGFR1, EGFR, and ROR2 polypeptides in MDA-MB-231 cells were analyzed by Western blotting. FGFR1, EGFR, and ROR2 polypeptides expressed in HEK293 cells were used as positive controls. GAPDH levels are displayed for normalization. The numbers below the FGFR1, EGFR, or ROR2 panel indicate the densitometric values of FGFR1, EGFR, or ROR2 bands relative to the corresponding GAPDH bands. (**B**) Serum-starved MDA-MB-231 cells with or without PTK7 knockdown were stimulated with 10 ng/mL aFGF or 10 ng/mL EGF for 5 min. The phosphorylation of FGFR1 and EGFR was analyzed in the growth-factor-stimulated MDA-MB-231 cells by Western blotting with pFGFR1 and pEGFR. GAPDH levels are displayed for normalization. Each bar represents the mean ± standard deviation (SD) from three independent experiments. * *p* < 0.05, ** *p* < 0.01, and *** *p* < 0.001 vs. growth-factor-stimulated cells transfected with control vector. ++ *p* < 0.01 and +++ *p* < 0.001 vs. cells without growth factor stimulation. # *p* < 0.05 vs. vector-transfected cells. n.s.: not significant.

The protein levels of FGFR1, EGFR, and ROR2, observed to bind to PTK7, were analyzed in MDA-MB-231 cells. It was found that FGFR1 and EGFR were expressed at high levels, whereas ROR2 expression was undetectable (Figure 6A). Subsequently, the effect of PTK7 knockdown on aFGF-induced FGFR1 phosphorylation and EGF-induced EGFR phosphorylation was examined in MDA-MB-231 cells, which showed the pronounced expression of FGFR1 and EGFR. Compared with control vectors, PTK7 knockdown significantly reduced the aFGF-induced phosphorylation of FGFR1 to 60.0 ± 8.0% and the EGF-induced phosphorylation of EGFR to 51.9 ± 17.9%, respectively (Figure 6B). The results demonstrated that PTK7 knockdown led to a reduction in both aFGF-induced FGFR1 phosphorylation and EGF-induced EGFR phosphorylation. In addition, PTK7 knockdown reduced the phosphorylation of FGFR1 induced by bFGF (Supplementary Figure S2A). Furthermore, PTK7 knockdown not only reduced aFGF-induced FRS2 phosphorylation (Supplementary Figure S2B), but also aFGF-induced activation of ERK and Akt (Supplementary Figure S2C). 

## 3. Discussion

Upregulation of PTK7 has been reported in many types of human malignancies [19]. In our previous study analyzing RNA-seq data from TCGA, the mean *PTK7* mRNA levels in endometrial, head and neck, lung, ovary, cervical, prostate, breast, pancreatic, bladder, thyroid, and esophageal cancer tissues were higher than the mean *PTK7* mRNA levels from all cancer tissues in the database [28]. Here, we showed that tumor tissues from BC patients had significantly higher *PTK7* mRNA levels than normal tissues. In TNBC patients, the *PTK7* mRNA level in tumor tissues was much higher than that in normal tissues. In addition, the PTK7 polypeptide was detected in all BC cell lines tested, including ER- and PR-positive BC cells and TNBC cells. This result was consistent with a prior report that PTK7 is highly expressed in TNBC cell lines [22]. Moreover, the expression of PTK7 in tumor-initiating cells from xenograft tissues derived from TNBC patients was higher than that in normal tissues [24]. Notably, it was reported that BC patients with low PTK7 expression had a more favorable prognosis than those with high PTK7 expression [22,33]. Based on these findings, we propose that PTK7 upregulation is a prospective prognostic determinant of BC, including TNBC.

PTK7 acts as an oncogene in different cell types. An increased expression of PTK7 correlates with poor clinical outcomes in acute myeloid leukemia, esophageal squamous cell carcinoma, colorectal cancer, cervical cancer, and thyroid cancer [14,17,34,35,36]. PTK7 is involved in cell proliferation, migration, and/or resistance to apoptosis in leukemia cells, colon cancer cells, liposarcoma cells, cervical cancer cells, and thyroid cancer cells [14,15,16,17,35,36]. Consistent with previous reports, we also found that the knockdown of PTK7 decreased the proliferation of six different BC cell lines derived from ER- and PR-positive BC and TNBC. In addition, knockdown of PTK7 inhibited the adhesion, migration, and invasion of three different TNBC cells. Epithelial–mesenchymal transition (EMT) is widely recognized as a crucial process in cancer cell migration and invasion [37]. Moreover, the involvement of PTK7 in EMT has been reported in hepatocellular carcinoma [38] and ovarian cancer [39]. Based on these findings, we hypothesized that PTK7 may also play a role in the migration and invasion of BC cells through EMT.

Although PTK7 lacks the catalytic activity of tyrosine kinase, PTK7 can induce oncogenic signals. We previously reported that PTK7 activates the Ras/MAP kinase and PI3K/Akt signaling pathways in ESCC cells [18]. ERK and JNK activate AP-1, and the PI3K–Akt–IKK signaling cascade activates NF-κB. AP-1 and NF-κB transactivate MMP-9 and enhance tumorigenic and invasive phenotypes. In addition, the impairment of PTK7 function inhibited the VEGF-induced activation of FAK and paxillin, and reduced cell migration and capillary-like tube formation in HUVECs [20]. However, it is known that oncogenic signaling pathways are highly activated in TNBC cells due to the frequent upregulation and activation of growth factors and RTKs [40,41,42,43,44]. For instance, MDA-MB-231 cells exhibit a high expression of EGF and EGFR [40,41], and MDA-MB-453 cells overexpress FGFR4 [42]. In the case of MDA-MB-436 cells, although the overexpression of RTKs was not known, they exhibit an overexpression of CXC chemokine receptor 4 (CXCR4) [43,44], a G-protein-coupled receptor known to play a crucial role in cell adhesion, survival, proliferation, and chemotaxis in different cell types. Despite their property of having high levels of oncogenic signaling, our findings indicate that the knockdown of PTK7 leads to a reduction in the phosphorylation of ERK, Akt, and FAK in TNBC cells.

It is known that catalytically inactive PTK7 can enhance the activation of catalytically active RTKs. PTK7 binds to and activates KDR molecules in endothelial cells, contributing to in vivo angiogenesis [21]. Additionally, PTK7 can activate FGFR1 by direct binding, independently of FGF, in ESCC cells [19]. Moreover, PTK7 plays a role in the activation of EGFR/Akt signaling in TNBC cells [32]. PTK7 is also known to interact with a catalytically inactive RTK, ROR2, and induce signaling pathways involved in cell migration [45]. PTK7 and ROR2 can interact with the Wnt5A ligand, thereby triggering non-canonical Wnt signaling in mammalian cells and during Xenopus embryo morphogenesis [46]. As reported, our pull-down analysis confirmed the interactions of PTK7 with ROR2, FGFR1, and EGFR. Notably, PTK7 exhibited strong binding to ROR2 and FGFR1, while the interaction with EGFR was comparatively weaker.

Previous studies have reported the differential expression of EGFR and FGFR1 in MDA-MB-231, MDA-MB-436, and MDA-MB-453 cells, with higher expression in MDA-MB-231 cells and lower expression in MDA-MB-436 and MDA-MB-453 cells [47,48]. Consistent with these reports, our findings confirm the high and moderate expression of EGFR and FGFR1 in MDA-MB-231 cells, respectively, while ROR2 was not detectable. Upon knockdown of PTK7, we observed a reduction in both aFGF/bFGF-induced FGFR1 phosphorylation and EGF-induced EGFR phosphorylation. In particular, PTK7 knockdown also reduced the aFGF-induced phosphorylation of FRS2 recruited by FGFR [49], and the phosphorylation of Akt and ERK, both of which are associated with downstream signaling pathways mediated by FGFR-FRS [50]. FGFR1 is highly expressed in BC tissues and cell lines, particularly in TNBC [51,52]. Therefore, it can be inferred that FGFR1 plays a key role in PTK7-induced Akt and ERK activation in MDA-MB-231 cells. Elevated levels of EGFR expression are associated with a more aggressive phenotype and poorer prognosis, especially in TNBC [53]. Additionally, EGFR has been implicated in the development of resistance to certain chemotherapy drugs [54]. Based on these observations, we propose that PTK7 enhances the oncogenic potential of TNBC through the activation of catalytically active RTKs, such as FGFR1 and EGFR. This is further supported by a recent study demonstrating the involvement of PTK7 in the activation of EGFR and Akt signaling pathways in TNBC cells [32].

Advances in therapeutic interventions tend to increase life expectancy after the onset of BC. However, TNBC has a high rate of metastasis and lacks ER, PR, and HER2, making treatment with hormone therapy and HER2-targeted therapy ineffective [2]. There is, therefore, a need for effective treatments of TNBC. Damelin et al. developed a PTK7-targeted antibody–drug conjugate (PTK7–ADC), and treatment with PTK7–ADC depleted tumor-initiating cells and induced tumor regression in TNBC-patient-derived or TNBC-cell-line-derived xenografts [24]. PTK7–ADC is cytotoxic to all cells expressing PTK7; therefore, it can have side effects. In this study, we found that PTK7 is oncogenic and activates PTK signaling. Agents that block the function of PTK7, such as a PTK7-neutralizing antibody, are likely to be an effective treatment with reduced side effects for PTK7-positive cancers such as BC, since they act only on cells in which PTK7 functions.

## 4. Materials and Methods

### 4.1. Analysis of PTK7 mRNA Levels in BC Tissues from the TCGA Database

A preprocessing RNA-seq count matrix and the associated clinical features of BC samples in the TCGA database were downloaded using the UCSC Xena browser (https://xenabrowser.net/ (accessed on 24 August 2020)) from the Genomic Data Commons (GDC; https://gdc.cancer.gov/ (accessed on 24 August 2020)). Gene expression was quantified as fragments per kilobase of transcript per million mapped reads upper quartile (FPKM-UQ), which is an RNA-seq-based expression-normalization method [55]. TNBC among the BC samples was defined as below 0 for Log_2_ (FPKM-UQ + 1) values of *ER*, *PR*, and *HER2* mRNA.

### 4.2. Cell Culture

Human BC cells were obtained from the Korean Cell Line Bank (Seoul, Republic of Korea). The human embryonic kidney (HEK) 293 cell line expressing the SV40 T antigen (HEK293T) was kindly provided by Professor Jang-Hee Hahn (Kangwon National University, Chuncheon, Republic of Korea). These cells were grown in Dulbecco’s modified Eagle medium (DMEM) supplemented with 5% fetal bovine serum (FBS) for BC cells, or 10% bovine serum for HEK293T cells, 100 U/mL penicillin, and 100 μg/mL streptomycin. All cells were grown at 37 °C in 5% CO_2_ and 95% air.

### 4.3. Western Blot Analysis

Cultured cells were lysed with RIPA lysis buffer (50 mM Tris-HCl, pH 7.4; 150 mM NaCl; 1% NP-40; 0.5% sodium deoxycholate; 0.1% SDS) supplemented with phosphatase inhibitors (5 mM NaF; 1 mM Na_3_VO_4_) for 10 min at 4 °C. Lysates containing protein were subjected to SDS–polyacrylamide gel electrophoresis (SDS-PAGE) and transferred onto a polyvinylidene fluoride membrane (Millipore, Bedford, MA, USA). Western blotting was performed using the following antibodies: anti-FLAG-M2 (F1804) obtained from Sigma-Aldrich (St. Louis, MO, USA); anti-HA (902302) obtained from BioLegend (San Diego, CA, USA); anti-phospho-ERK (sc-7383) and anti-FAK (sc-557), obtained from Santa Cruz Biotechnology (Santa Cruz, CA, USA); anti-phospho-Akt (Ser473, 4060s), anti-Akt (9272s), anti-phospho-EGFR (Tyr1068, 3777s), anti-EGFR (2232s), anti-phospho-FGFR (Tyr653/654, 3471s), anti-phospho-FRS2-alpha (Tyr436, 3861s), and anti-ROR2 (88639s), obtained from Cell Signaling Technology (Danvers, MA, USA); anti-FRS2 (ab183492), obtained from Abcam (Cambridge, UK); anti-phospho-FAK (Tyr397, abt135), obtained from Merck Millipore (Burlington, MA, USA); anti-ERK2 (bms-52068R), obtained from Bioss (Boston, MA, USA); anti-GAPDH (abc2003), obtained from AbClone (Seoul, Republic of Korea); and horseradish-peroxidase-conjugated goat anti-mouse IgG (K0211589) and rabbit IgG (K0211708), obtained from KOMA Biotech (Seoul, Republic of Korea). Rabbit anti-PTK7 anti-serum, which recognizes both human and murine PTK7, was described previously [20]. Immune reactions were visualized using Immobilon Western Chemiluminescent HRP Substrate (Millipore) and an Amersham ImageQuant 800 (Cytiva, Marlborough, MA, USA) imaging system.

### 4.4. Generation of PTK7-Knockdown Lentivirus and Infection of BC Cells

Lentiviruses were produced in HEK293T cells co-transfected with 10 μg of pLKO.1-control or pLKO.1-shRNA-PTK7-6434 plasmid, 7.4 μg of packaging vector psPAX2, and 2.6 μg of envelope vector pMD2.G, using the calcium-phosphate method [56]. After overnight incubation, the medium was replaced, and the cells were incubated for an additional 48 h. Medium containing lentivirus was filtered through a 0.45 μm membrane filter. BC cells were then infected by incubation with a 1:1 mixture of lentivirus-containing supernatant and fresh medium containing 8 μg/mL polybrene for 24 h, after which the medium was replaced with fresh medium. The cells were incubated for 24 h and then cultured with 2.5 μg/mL puromycin for 14 days, whereby puromycin-resistant colonies were produced as a mixed culture.

### 4.5. Cell Proliferation Assay

The cell proliferation assay was performed as previously described [57]. The subconfluent cells were detached and resuspended in fresh medium. Cells (4 × 10^3^/well) were seeded in 96-well plates and incubated with 0.1 mL DMEM supplemented with 5% FBS. At the specified time intervals, cells were incubated with 0.1 mL DMEM containing 0.5 mg/mL MTT in a 5% CO_2_ incubator at 37 °C for 4 h. After incubation, the cells were washed twice with PBS and the formazan reaction product was dissolved by adding 0.1 mL of DMSO. The absorbance of the colored solution was measured at 565 nm.

### 4.6. Cell Adhesion Assay

The cell adhesion assay was performed as previously described [57]. Briefly, detached cell suspensions (1 × 10^4^ cells/0.1 mL) were loaded onto 96-well plates coated with rat tail type I collagen (1 μg/well) or fibronectin (1 μg/well) and incubated for 2 h. The cells were then fixed with 3.7% paraformaldehyde in PBS and stained with 0.005% crystal violet. The stained cells were lysed with 1% SDS and the absorbance was measured at 600 nm.

### 4.7. Chemotactic Migration Assay

Chemotactic migration assays were performed as previously described [58]. Briefly, detached cell suspensions (1 × 10^5^ cells/0.2 mL) were loaded into the upper chamber of Transwell filters (8 μm pore size, Corning, Corning, NY, USA). The lower surface of each Transwell was coated with 10 μL of 0.1% gelatin and dried for 30 min at room temperature. The lower compartment of each well was filled with 0.6 mL DMEM with 10% FBS as a chemoattractant. The chamber was incubated at 37 °C for 24 h. Cells that migrated to the lower surface of the filter were fixed with 3.7% paraformaldehyde in PBS for 15 min and stained with 0.005% crystal violet for 5 min. After removing the remaining cells on the upper surface of the upper chamber with a cotton swab, the stained cells were solubilized with 1% SDS, and the absorbance was measured at 600 nm.

### 4.8. Invasion Assay

The invasion assay was performed using a Transwell system as previously described [19]. The lower side of the upper chamber was coated with 10 μL of 0.1% gelatin and dried for 30 min at room temperature. The upper surface of the filter was coated with 80 μL of 0.3 μg/mL growth-factor-reduced Matrigel (Corning) and dried overnight. The detached cells were washed and resuspended in a serum-free medium. The cell suspension (0.1 mL, 5 × 10^5^ cells/mL) was loaded into the upper chamber and 0.6 mL of DMEM supplemented with 5% FBS as a chemoattractant was added to the lower chamber. The chamber was incubated for 24 h at 37 °C. The staining and measurement of the cells which had invaded were the same as those used in the chemotactic migration assay.

### 4.9. Constructs Expressing PTK7-FLAG, FGFR1-HA, EGFR-HA, and ROR2-HA

pcDNA3-PTK7-FLAG encoding human PTK7 with a C-terminal FLAG-tag and pcDNA3.1-FGFR1-HA encoding human FGFR1 with C-terminal HA-tag has previously been described [19]. pcDNA3.1-EGFR-HA encoding full-length human EGFR with a C-terminal HA-tag was constructed by PCR amplification and subcloning. The template was pcDNA3.1-FLAG-EGFR harboring human full-length EGFR cDNA [59,60]. The primer pairs were 5′-ACCCAAGCTG*GCTAGC*ACC**ATG**CGACCCTCCGGGACGG-3′, including a NheI site (italicized), a Kozak sequence (underlined), and nt. 262–280 of GenBank NM_005228, including a start codon (bold), and 5′-GCCCTCTAGA*CTCGAG***TTA**AGCGTAATCTGGAACATCGTATGGGTATGCTCCAATAAATTCACTGCTTTGTGGCGC-3′, including a XhoI site (italicized), a stop codon (bold), HA-tagged coding sequence (underlined), and nt. 3894–3862 of GenBank NM_005228. The PCR product was ligated into the NheI-XhoI sites of the pcDNA3.1 vector (Thermo Fisher Scientific, Waltham, MA, USA). pcDNA3.1-ROR2-HA encoding full-length human ROR2 with a C-terminal HA-tag was also constructed by PCR amplification and subcloning. The template was pCR4-TOPO-ROR2 containing human full-length ROR2 cDNA (Addgene, Watertown, MA, USA). The primer pairs were 5′-GC*GCTAGC*CACC**ATG**GCCCGGGGCTCG-3′, including a NheI site (italicized), a Kozak sequence (underlined), and nt. 88–102 of GenBank BC130522, including a start codon (bold), and 5′-CG*TCTAGA***TTA**AGCGTAATCTGGAACATCGTATGGGTAAGCTTCCAGCTGGACTTGGGC-3′, including a XbaI site (italicized), a stop codon (bold), HA-tagged coding sequence (underlined), and nt. 2917–2896 of GenBank BC130522. The PCR product was ligated into the NheI-XbaI sites of the pcDNA3.1 vector (Thermo Fisher Scientific). Final constructs were confirmed to have no PCR errors by performing DNA sequencing.

### 4.10. Binding Analysis of PTK7 with FGFR1, EGFR, and ROR2

HEK293 cells were transfected with pcDNA3-PTK7-FLAG and pcDNA3.1-FGFR1-HA, pcDNA3.1-EGFR-HA, or pcDNA3.1-ROR2-HA, encoding human FGFR1, EGFR, or ROR2, with a C-terminal HA tag, respectively, by calcium phosphate method [56]. Subconfluent HEK293 cells co-expressing PTK7-FLAG and FGFR1-HA, EGFR-HA, or ROR2-HA were lysed with NP-40 lysis buffer (50 mM Tris–HCl, pH 7.4, 150 mM NaCl, and 1% NP-40) containing 5 mM NaF, 1 mM Na_3_VO4, and protease inhibitor cocktail III (Calbiochem, La Jolla, CA, USA). The lysates were incubated with mouse anti-FLAG M2-agarose (Sigma-Aldrich) for 2 h. The protein-bound resins were then washed with NP-40 lysis buffer. Pulled-down proteins were resuspended in SDS sample buffer and subjected to Western blotting.

### 4.11. Statistical Analysis

All data were obtained from at least three independent experiments and are expressed as the mean ± standard deviation. Statistical significance was analyzed using Student’s *t*-tests. A *p*-value less than 0.05 was considered statistically significant.

## 5. Conclusions

Despite being catalytically defective, PTK7 is expressed in various carcinoma types, particularly in BC and TNBC. We observed that knockdown of PTK7 significantly reduced proliferation in BC cells, including TNBC cells, as well as decreased adhesion, migration, and invasion in TNBC cells. In addition, PTK7 knockdown decreased the phosphorylation of ERK, AKT, and FAK in TNBC cells, which are key regulators for cell proliferation, migration, and invasion. PTK7 was found to interact with FGFR1, EGFR, and ROR2, with a particularly strong binding to ROR2 and FGFR1. In MDA-MB-231 cells, in which FGFR1 and EGFR were highly expressed and ROR2 was not expressed, knockdown of PTK7 led to a reduction in the phosphorylation of FGFR1 and EGFR. This suggests that PTK7 plays a role in regulating the phosphorylation of FGFR1 and EGFR by binding to these RTKs. Consequently, PTK7 emerges as a pivotal factor in the tumorigenesis and metastasis of BC, positioning it as a promising therapeutic target for BC, including TNBC.

## Data Availability

Data are contained within the article.

## Supplementary Materials

The supporting information can be downloaded at: https://www.mdpi.com/article/10.3390/ijms241512173/s1.
