# Supplementary material for: Knockdown of PTK7 Reduces the Oncogenic Potential of Breast Cancer Cells by Impeding Receptor Tyrosine Kinase Signaling
Source: Int J Mol Sci. 2023 Jul 29;24(15):12173. doi: 10.3390/ijms241512173 (PMC10418930; doi:10.3390/ijms241512173)
Supplement: Supplementary file 1 [file ijms-24-12173-s001.zip › ijms-2492870-supplementary.pdf]

Supplementary Materials

Knockdown of PTK7 reduces the oncogenic potential of breast cancer cells by impeding receptor tyrosine kinase signaling

Won-Sik Shin[1,]+, Si Won Oh[1,]+, Han Na Park[1], Jae Hoon Kim[1], and Seung-Taek Lee[1,]*

[1] Department of Biochemistry, College of Life Science and Biotechnology, Yonsei University, Seoul 03722, Republic of Korea; amgod0306@hanmail.net (W.S.S.); siwon1993@gmail.com (S.W.O.); qkrgkssk1024@naver.com (H.N.P.); ebahspa@yonsei.ac.kr (J.H.K.)

* Correspondence: stlee@yonsei.ac.kr; Tel.: +82-2-2123-2703

+ These authors contributed equally to this work.

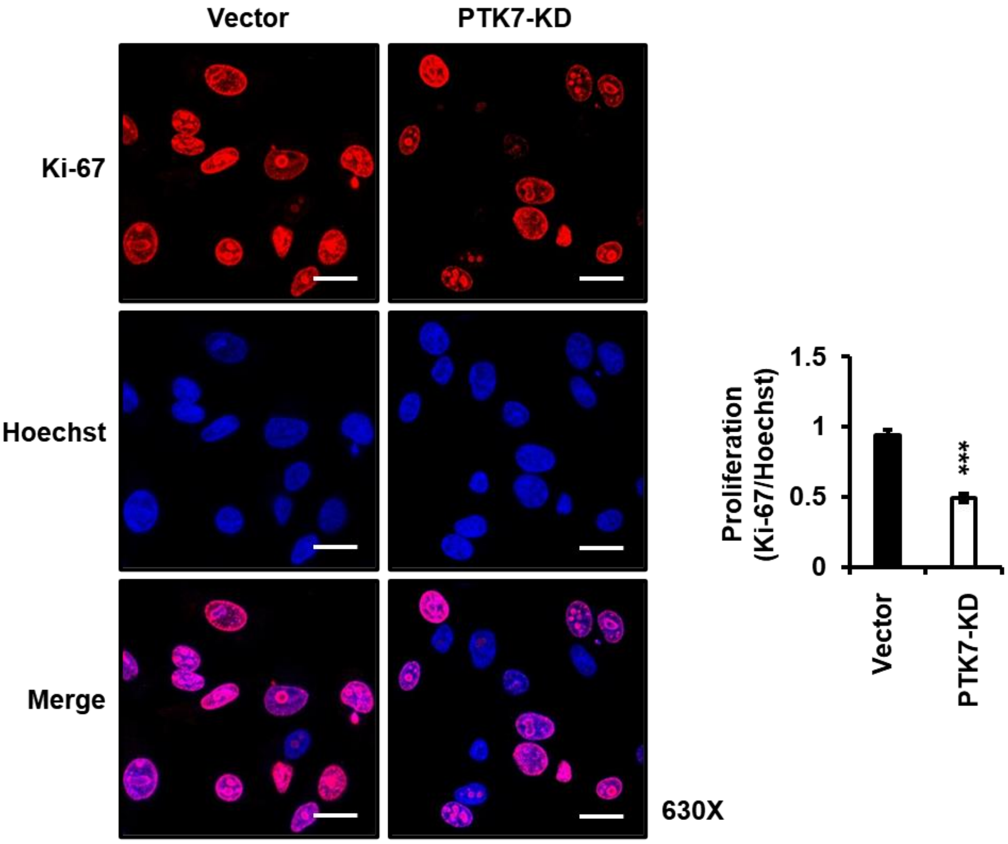

Supplementary Figure S1. Effect of PTK7 knockdown on Ki-67 levels in MDA-MB-231 cells.

MDA-MB-231 cells plated on confocal dish (Corning) were incubated for 24 h in DMEM with 5% fetal bovine serum. The cells were fixed with 3.7% paraformaldehyde for 15 min and permeabilized in 0.2% Triton-X 100 for 10 min. Cells were blocked in 3% bovine serum albumin for 30 min and immunostained overnight at 4°C with 2.5 μg/mL of anti-Ki-67 antibody (8D5; Cell Signaling Technology) to stain proliferating cells. The cells were washed with phosphate-buffered saline and incubated with 1 U/ml of Rhodamine Red-X-conjugated goat anti-mouse IgG (H + L) antibody (Thermo Fisher Scientific) for 2 h. Additionally, Hoechst 33258 at a concentration of 2 μg/mL was applied for 30 min to stain cell nuclei. Images were obtained using confocal microscope (LSM880; Carl Zeiss) with 63X Plan-Apochromat objective lens and the Zen software (version 3.491; Carl Zeiss). The excitation wavelengths were 405 nm for Hoechst 33258 and 555 nm for Rhodamine Red-X. To ensure unbiased image acquisition, all images were obtained from randomly selected fields using consistent parameters, including exposure time, laser power, and offset setting. The fluorescence intensity was quantified using ImageJ software. Representative micrograph images (630X) and a graph illustrating the relative level of Ki-67 staining to Hoechst 33258 staining were presented. Each value represents the mean ± SD of three independent experiments. *** $P <$ 0.001 vs. Vector. Scale bar = 200 μm.

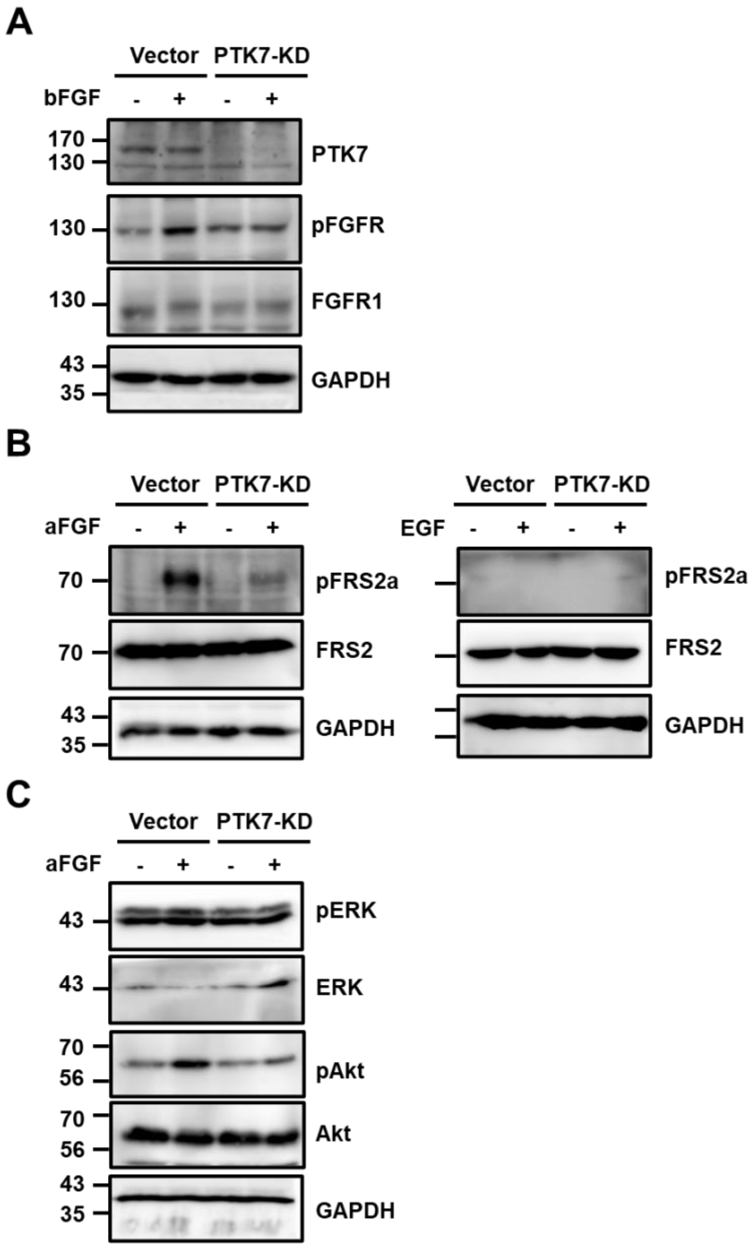

Supplementary Figure S2. Effect of PTK7 knockdown on the phosphorylation of FGFR1 and downstream signaling proteins in MDA-MB-231 cells.

(**A**) Serum-starved MDA-MB-231 cells with or without PTK7 knockdown were stimulated with 10 ng/ml bFGF for 5 min. Phosphorylation of FGFR1 was analyzed by western blotting with pFGFR. (**B** and **C**) Serum-starved MDA-MB-231 cells with or without PTK7 knockdown were stimulated with 10 ng/ml aFGF or 10 ng/ml EGF for 5 min. Phosphorylation of FRS2a was analyzed in the growth factor-stimulated MDA-MB-231 cells by western blotting with pFRS2a (**B**). Phosphorylation of ERK and Akt was analyzed in the aFGF-stimulated MDA-MB-231 cells by western blotting (**C**). GAPDH levels are displayed for normalization.
